# Supplementary material for: Association Between Maternal Diet and Frequency of Micronuclei in Mothers and Newborns: A Systematic Review
Source: Nutrients. 2025 Aug 1;17(15):2535. doi: 10.3390/nu17152535 (PMC12348456; doi:10.3390/nu17152535)
Supplement: Supplementary file 1 [file nutrients-17-02535-s001.zip › nutrients-3781185-supplementary.pdf]

Supplementary File – Table S1

Results of the application of the search strategy in the databases;

Bases	Strategy	Results
Embase	(('pregnancy'/exp OR pregnancy OR pregnancy:ti,ab,kw OR 'pregnant woman':ti,ab,kw OR newborn:ti,ab,kw OR 'mother child relation':ti,ab,kw) AND 'maternal exposure':ti,ab,kw OR 'maternal nutrition':ti,ab,kw OR diet:ti,ab,kw OR nutrient:ti,ab,kw) AND 'micronucleus test':ti,ab,kw	104
Pubmed	(Pregnancy [MESH terms] OR Gestation OR Pregnant Woman [MESH terms] OR Woman, Pregnant Women OR Pregnant OR INFANT [MESH] (Infants) Newborn [MESH terms] OR Newborn Infants OR Newborn Infant OR Newborns OR Neonate OR Neonates OR Mother-Child Relations [MESH]) AND (Maternal Exposure [MESH terms] OR Exposure, Maternal OR Maternal Exposures OR Diet [MESH terms] OR Maternal Diet OR Maternal Nutrition OR Nutrients [MESH terms] OR Nutrient OR Macronutrients OR Micronutrients) AND (Micronucleus Tests [MESH terms] OR Micronucleus Test OR Micronuclei, Chromosome-Defective [MESH terms] OR Micronucleus frequencies OR DNA damage [MESH terms])	200
Science direct	(Pregnancy [MESH terms] OR Newborn [MESH terms] OR Mother-Child Relations [MESH]) AND (Maternal Exposure [MESH terms] OR Diet [MESH terms] OR Maternal Nutrition) AND (Micronucleus Tests [MESH terms] OR Micronuclei frequency OR Micronuclei, Chromosome-Defective [MESH terms])	202
Scopus	(TITLE-ABS-KEY (pregnancy) OR TITLE-ABSKEY (gestation) OR TITLE-ABS-KEY ("pregnant woman") OR TITLEABS-KEY (pregnant) OR TITLE-ABS-KEY (infant) OR TITLE-ABSKEY (newborn) OR TITLE-ABS-KEY ("newborn infants") OR TITLEABS-KEY ("newborn infant") OR TITLE-ABSKEY (newborns) OR TITLE-ABS-KEY (neonate) OR TITLE-ABSKEY (neonates) OR TITLE-ABS-KEY ("mother-child relations") AND TITLE-ABS-KEY ("maternal exposure") OR TITLE-ABSKEY (diet) OR TITLE-ABS-KEY ("maternal diet") OR TITLE-ABSKEY ("maternal nutrition") OR TITLE-ABS-KEY (nutrients) OR TITLEABS-KEY (nutrient) OR TITLE-ABS-KEY (macronutrients) OR TITLEABS-KEY (micronutrients) AND TITLE-ABS-KEY ("micronucleus tests") OR TITLE-ABS-KEY ("micronucleus test") OR TITLE-ABSKEY ("micronuclei, chromosome-defective") OR TITLE-ABSKEY ("micronucleus frequencies"))	61
Web of science	(((((((((((ALL=(Pregnancy)) OR ALL=(Gestation)) OR ALL=(Pregnant Woman)) OR ALL=(Woman, Pregnant Women)) OR ALL=(Pregnant)) OR ALL=(INFANT)) OR ALL=(Newborn)) OR ALL=(Newborn Infants)) OR ALL=(Newborn Infant)) OR ALL=(Newborns)) OR ALL=(Neonate)) OR ALL=(Neonates)) OR ALL=(Mother-Child Relations)<br>(((((((((ALL=(Maternal Exposure)) OR ALL=(Exposure, Maternal)) OR ALL=(Maternal Exposures)) OR ALL=(Diet)) OR ALL=(Maternal Diet)) OR ALL=(Maternal Nutrition)) OR ALL=(Nutrients)) OR ALL=(Nutrient)) OR ALL=(Macronutrients)) OR ALL=(Micronutrients)<br>((((ALL=(Micronucleus Tests)) OR ALL=(Micronucleus Test)) OR ALL=(Micronuclei, Chromosome-Defective)) OR ALL=(Micronucleus frequencies)) OR ALL=(DNA damage)	651
Google academico	(Pregnancy OR Mother-newborns OR Newborn) AND (Dietary estimates OR Maternal diet) AND (Micronuclei frequency OR Micronucleus frequencies)	200

Supplementary File – Table S2

Excluded articles and justifications.

Articles	Reasons for deletion
DNA damage analysis in newborns and their mothers related to pregnancy and delivery characteristics	This article used "nutritional history"

The Impact of Mother's Living Environment Exposure on Genome Damage, Immunological Status, and Sex Hormone Levels in Newborns	The food questionnaire was applied after delivery.
Increased micronuclei and bulky DNA adducts in cord blood after maternal exposures to traffic-related air pollution	Did not present the results of the relationship between MN and diet
Micronuclei levels in mothers and their newborns from regions with different types of air pollution	This study did not evaluate the diet consumed
Environmental, Dietary, Maternal, and Fetal Predictors of Bulky DNA Adducts in Cord Blood: A European Mother–Child Study (NewGeneris)	This study did not evaluate the frequency of micronuclei
NewGeneris: a European study on maternal diet during pregnancy and child health	Protocol article/literature review
Bulky DNA Adducts in Cord Blood, Maternal Fruit-and-Vegetable Consumption, and Birth Weight in a European Mother–Child Study (NewGeneris)	This study did not evaluate the frequency of micronuclei
Micronucleus frequency in human umbilical cord lymphocytes	This study did not evaluate the diet consumed
Micronuclei in cord blood lymphocytes as a biomarker of transplacental exposure to environmental pollutants	This study did not evaluate the diet consumed
Maternal and Gestational Factors and Micronucleus Frequencies in Umbilical Blood: The NewGeneris Rhea Cohort in Crete	Did not present the results of the relationship between MN and diet
Infant birth outcomes are associated with DNA damage biomarkers as measured by the cytokinesis block micronucleus cytome assay: the DADHI study	The food questionnaire was applied after delivery, at 3 and 6 months. This study did not evaluate the diet consumed
Increased lymphocyte micronucleus frequency in early pregnancy is associated prospectively with pre-eclampsia and/or intrauterine growth restriction	Study not performed with pregnant women
Folate, Vitamin B12, Vitamin B6 and homocysteine: impact on pregnancy outcome	Did not present the results of the relationship between MN and diet

High Dietary Folic Acid Intake Is Associated with Genomic Instability in Peripheral Lymphocytes of Healthy Adults	This study did not evaluate the frequency of micronuclei
Effects of Benzo[a]pyrene-DNA adducts, dietary vitamins, folate, and carotene intakes on preterm birth: a nested case-control study from the birth cohort in China	This study did not evaluate the diet consumed
Dioxin-like exposures and effects on estrogenic and androgenic exposures and micronuclei frequency in mother-newborn pairs	This study did not evaluate the frequency of micronuclei nor the diet consumed
Chromosomal aberrations in cord blood are associated with prenatal exposure to carcinogenic polycyclic aromatic hydrocarbons	This article used the "food diary"
The interaction between air pollution and diet does not influence the DNA damage in lymphocytes of pregnant women	This study did not evaluate the frequency of micronuclei
Dietary and Antioxidant Vitamins Limit the DNA Damage Mediated by Oxidative Stress in the Mother-Newborn Binomial	This study did not evaluate the frequency of micronuclei
Dietary Patterns in Pregnancy and Biomarkers of Oxidative Stress in Mothers and Offspring: The NELA Birth Cohort	This study did not evaluate the frequency of micronuclei
Increased frequency of micronuclei in mononucleated lymphocytes and cytome analysis in healthy newborns as an early warning biomarkers of possible future health risks	This study did not evaluate the diet consumed
Evaluation of the genotoxicity of 10 selected dietary/environmental compounds with the in vitro micronucleus cytokinesis-block assay in an interlaboratory comparison	Study not performed with pregnant women
The use of genotoxicity biomarkers in molecular epidemiology: applications in environmental, occupational and dietary studies	Literature review article
Prenatal, early life, and childhood exposure to genotoxicants in the living environment	Literature review article

Supplementary File – Table S3

Assessment of methodological quality and characteristics of studies included in the review.

Cohort – Newcastle-Ottawa Scale

	Selection				Comparability	Outcome			
**Quality criteria**	**1 Representativeness of the exposed cohort**	**2 Selection of the non-exposed cohort**	**3 Ascertainment of exposure**	**4 Demonstration that outcome of interest was not present at start of study**	**1 Comparability of cohorts on the basis of the design or analysis**	**1 Assessment of outcome**	**2 Was follow-up long enough for outcomes to occur?**	**3 Adequacy of follow-up of cohorts**	**Total Score**
O'CallanghanGordo et al. (2015)		☆	☆	☆	☆	☆	☆		6
Loock et al. (2014)		☆	☆	☆	☆	☆			5
O'CallanghanGordo et al. (2017)		☆	☆	☆	☆	☆	☆	☆	7
O'CallanghanGordo et al. (2018)		☆	☆	☆	☆	☆	☆		6

Cross-sectional Newcastle-Ottawa Scale Modified

	Selection				Comparability	Outcome		
**Quality criteria**	**1 Representativeness of the sample**	**2 Sample size**	**3 Ascertainment of exposure**	**4 Non-responders**	**1 The subjects in different groups of results are compatible based on the study design and in the analysis. The base factors are controlled.**	**1 Evaluation of the results**	**2 Statistical test**	**Total Score**
Pedersen et al. (2012)				☆	☆☆	☆	☆	5
